# Does protamine infusion reduce postoperative blood loss and heparin rebound in cardiac surgery patients? A retrospective analysis of 240 patients on the cardiac ICU

**DOI:** 10.1186/cc12301

**Published:** 2013-03-19

**Authors:** E Helme, H Meeran, N Al-Subaie

**Affiliations:** 1 St George's Hospital, London, UK

## Introduction

Cardiac surgery is associated with significant blood loss. Teoh and colleagues demonstrated a reduction in postoperative bleeding with the use of a protamine infusion and an abolishment of heparin rebound [1]. The aim of this study was to see whether the use of postoperative protamine infusions in our cardiac ITU was associated with a reduction in heparin rebound and blood loss.

## Methods

Data from 240 cardiac surgery patients were retrospectively analysed. Of these, 157 had routine management with a bolus of protamine to correct the activated clotting time and then expectant management of subsequent bleeding, and 47 had the same but also a protamine infusion of 10 to 80 mg/hour for between 3 and 8 hours postoperatively. Blood loss was measured at 1, 6, 12 and 24 hours. In all, excessive bleeding was investigated using thromboelastography (TEG). Rebound heparinisation was determined by a ratio of R-times (heparinase/plain) <0.8. The Mann-Whitney U test and the chi-squared test were used to assess statistical significance.

## Results

There was no significant difference in blood loss between the two groups. Blood loss at 1 hour in the infusion and non-infusion group was 145 and 88 ml, respectively (*P *= 0.06); at 6 hours: 450 and 392 ml (*P *= 0.5); at 12 hours: 620 and 595 ml (*P *= 0.62); and at 24 hours: 971 and 872 ml (*P *= 0.12). There was also no significant difference in those getting heparin rebound with 40% in the infusion group and 47% in the non-infusion group (*P *= 0.54).

## Conclusion

Unlike Teoh and colleagues [1], we did not find an advantage in using protamine infusions. That there were still cases of heparin rebound in the infusion group suggests that the infusion was not as effective as expected and/or the dose was inadequate. However, previous studies assessed heparin rebound using isolated clotting parameters [1,2]. Here, we used TEG. As TEG measures the thrombodynamic properties of whole blood coagulation, perhaps it is a more reliable indicator of heparin activity? As a retrospective study, there are limitations; namely, the nonstandardised management of the patients and the potential bias in the anaesthetists' selection of patients for an infusion. This group may be inherently higher risk for bleeding. However, heparin rebound is common and protamine is a simple, relatively safe and low-cost intervention compared with transfusion and so further study is needed.
